# Addressing Molecular Diagnosis of Occupational Allergies

**DOI:** 10.1007/s11882-018-0759-9

**Published:** 2018-02-14

**Authors:** Monika Raulf, Santiago Quirce, Olivier Vandenplas

**Affiliations:** 1 Institute of Prevention and Occupational Medicine of the German Social accident Insurance, Institute of the Ruhr-University Bochum (IPA), Bochum, Germany; 2 Department of Allergy, Hospital La Paz Institute for Health Research (IdiPAZ) and CIBER of Respiratory diseases (CIBERES), Madrid, Spain; 3 Centre Hospitalier Universitaire UCL Namur, Department of Chest Medicine, Université Catholique de Louvain, Yvoir, Belgium

**Keywords:** Baker’s asthma, Component-resolved diagnosis, IgE determination, Natural rubber latex, Occupational allergy, Wheat allergens

## Abstract

**Purpose of Review:**

Numerous clinically relevant allergenic molecules enhance the performance of specific (s) IgE tests and improve the specificity of allergy diagnosis. This review aimed to summarize our current knowledge of the high-molecular-weight allergens involved in the development of occupational asthma and rhinitis and to critically analyze the contribution of component-resolved diagnosis in the management of these conditions.

**Recent Findings:**

There is a lack of standardization and validation for most available extracts of occupational agents, and assessment of sIgE reactivity to occupational allergen components has been poorly investigated, with the notable exception of natural rubber latex (NRL) and wheat flour. In the case of NRL, the application of recombinant single allergens and amplification of natural extracts with stable recombinant allergens improved the test sensitivity. IgE-sensitization profile in patients with baker’s asthma showed great interindividual variation, and extract-based diagnostic is still recommended. For other occupational allergens, it remains necessary to evaluate the relevance of single allergen molecules for the sensitization induced by occupational exposure.

**Summary:**

Progress has been made to characterize occupational allergens especially NRL and wheat, although there is still an unmet need to increase the knowledge of occupational allergens, to include standardized tools into routine diagnostic, and to evaluate their usefulness in clinical practice.

## Introduction

Sensitizer-induced occupational asthma (OA) and rhinitis (OR) are associated with a substantial public health burden because of their high prevalence and socio-economic impacts [1, 2•]. More than 400 occupational agents are currently documented in the scientific literature as being potential “respiratory sensitizers” [3, 4, 5••]. These agents that may induce OR or OA can be categorized into high-molecular-weight (HMW) and low-molecular-weight (LMW) agents. The mechanism by which HMW agents, which are proteins or glycoproteins, induce OR or OA is thought to be IgE-mediated similar to that known to cause allergic rhinitis or allergic asthma induced by ubiquitous allergens in the general population. The most common occupational HMW agents are proteins or glycoproteins found in flour and grains, enzymes, laboratory animals, fish and seafood, fodder and detergent enzymes, molds (fungi), and *Hevea brasiliensis* latex. Typical LMW substances are isocyanates, persulfate salts, metals, quaternary ammonium persulfate, acid anhydrides, cleaning products/disinfectants, and medicinal drugs. The pathogenesis of OR or OA caused by LMW agents remains poorly understood but may include both immunologic and non-immunologic mechanisms. An IgE-mediated mechanism has been documented for a few LMW agents such as anhydride acids, platinum salts, and reactive dyes. It is generally assumed that the allergenicity of these LMW or their metabolites result from a mostly covalent interaction with some carrier proteins to build a hapten-carrier complex [6, 7].

The diagnosis of OA and OR diseases most often remains a challenge [8••]. Only few diagnostic methods and occupational allergen extracts or substances are available in a standardized form, because the respiratory-sensitizing properties of most occupational substances have been documented only as individual case reports. The basis for accurate diagnosis, management, and prevention is the deeper knowledge of the allergic properties of the hazards and suitable diagnostic test methods as well as the identification of individual and occupational risk factors.

The differential diagnosis of work-related asthma should be made on objective basis using an algorithm of clinical, physiologic, and immunological testing (e.g., skin prick tests (SPT), determination of specific IgE antibodies, and assessment of inflammatory biomarkers) [2•]. A careful assessment of the clinical history is of great importance for the interpretation of the findings (like SPT or in vitro tests). Determination of specific IgE (sIgE) antibodies and SPT are both considered equally effective in the diagnosis of allergy, but both tests show only the presence of a specific IgE-mediated sensitization to an agent present at the workplace. The causal relationship between exposure and symptoms can usually be ascertained by the specific inhalation challenge test. In practice, SPT is often taken as the method of choice because results are available immediately and the procedure is cost effective. It should be noted that a positive SPT does not necessarily correlate with the presence of sIgE (and vice versa). Unfortunately, there is a lack of standardization and validation for most available extracts of occupational agents and the allergenic potency of SPT extracts may vary significantly among manufacturers. Comparison of wheat and rye SPT solutions for diagnosis of baker’s asthma from three companies showed differences in protein concentrations and compositions with consequences of widely differing SPT results. Using allergen-specific bronchial challenge as a gold standard, the sensitivity of SPTs was between 40 and 67%, specificity was between 86 and 100%, and the positive predictive value ranged from 81 to 100% and negative predictive value from 44 to 70% [9]. A multi-center study to evaluate SPT solutions for selected occupational allergens confirmed that there is a wide variability of SPT solutions for protein and antigen content, and the sensitivity of several solutions may be low. Thus, improvement and standardization of SPT solutions for occupational allergens is essential [10, 11]. A meta-analysis of studies including subjects exposed to various HMW agents found that SPT and sIgE determination provided sensitivity rates of 81% (95% CI 70–88%) and 73% (95% CI 64–81%), respectively, as compared to specific inhalation challenge, while the specificity of these tests was 60% (95% CI 42–75%) and 79% (95% CI 50–93%) [12].

IgE antibodies are a marker of immediate hypersensitivity, and therefore the determination of allergen-sIgE in serum is undoubtedly the most important in vitro test. Assays such as commercial solid-phase systems (e.g., ImmunoCAP [ThermoFisher/Phadia, Uppsala, Sweden]) or in-house ELISA tests and immunoblotting can be used for the detection and/or quantification of sIgE antibodies. Crude extracts from the different allergen sources have been traditionally used for the detection of sIgE. Similar to SPT, the composition and amount of an allergenic extract strongly affect the results [10, 11, 13•]. Therefore, the results of IgE immunoassays from different companies are often not comparable and sometimes produced non-concordant qualitative results and potentially leading to misjudgment of sensitization. Although under certain circumstances, high level of sIgE (the same is true for SPT results) allows for predicting a positive response to the inhalation challenge test with a high level of confidence, especially in symptomatic bakers and health care workers exposed to NRL [14, 15], these threshold values are cohort-specific and should be extrapolated with caution to individual patients [16]. Nevertheless, there is accumulating evidence that patients with high sIgE will develop a positive challenge response to the relevant allergen.

Component-resolved diagnosis (CRD) is based on the assessment of sIgE using single allergenic components purified from natural sources or produced by recombinant techniques. A prerequisite for CRD is the characterization of the protein components of the allergens [17]. Allergenic molecules are analyzed individually or can be used to supplement labile or missing allergens in crude extracts (e.g., spiking of allergen extracts with single allergen molecules). In general, the transition from allergen extracts to molecules and CRD for the diagnostic work-up of IgE-mediated reactions has great potential and offers the possibility to discriminate between genuine allergy and merely sensitization to establish individual sensitization patterns and to predict the individual risk of severe allergic reactions. However, detailed knowledge of the various allergen molecules, protein families, and cross-reactivity is absolutely required for an accurate and clinically relevant interpretation of the results. Therefore, the European Academy of Allergy and Clinical Immunology (EAACI) edited in 2016 the “Molecular Allergology User’s Guide” to summarize and spread all these necessary information to health professionals who are dealing everyday with allergic patients [18••].

Nevertheless, only few of the HMW occupational allergens are characterized and/or produced in recombinant form and commercially available for the in vitro diagnosis. This review aimed at summarizing our current knowledge of the HMW allergens involved in the development of OA and OR and to critically analyze the contribution of CRD in the diagnosis and management of these conditions.

## Natural Rubber Latex

In the 1980s, NRL allergy emerged as an epidemic in health care facilities where powdered NRL gloves were used. Exposed workers and also patients, especially those with frequent surgeries in their early life (e.g., patients with spina bifida), suffered from urticaria, rhinitis, asthma, and/or anaphylaxis. Based on the worldwide attention of this health problem and international collaborations of researchers, occupational hygienists, government agencies, manufacturers, and health policy makers, NRL allergy is not only nearly eliminated in industrialized countries, but *Hevea brasiliensis*, the origin of NRL, is the best-characterized occupational allergen source [19, 20, 21••]. Up to now, 15 NRL allergens are identified and included in the official allergen list of the World Health Organization and International Nomenclature Union of Immunological Societies (WHO/IUIS) Allergen Nomenclature database and assigned official numbers Hev b 1–15 (http://www.allergen.org) (Table 1). Several recombinant Hev b-allergens are available as singleplex [22] or multiplex tool [23–25]. They are useful for the determination of sensitization profiles. In plant allergen such as NRL, the presence of cross-reactive carbohydrate determinants (CCDs) can negatively influence the assessment of sIgE antibodies. Therefore, it is necessary to exclude glyco-epitopes with low clinical relevance that may be responsible for IgE-binding. Attention should be paid to this CCD-based cross-reactivity by using corresponding CCD screening tools (e.g., horseradish peroxidase, bromelain, ascorbate oxidase) and/or inhibition testing. In a recent paper, Hemmer et al. [26••] demonstrated that cellulose used as solid-phase allergen carrier (e.g., ThermoFisher/Phadia ImmunoCAP platform) can contain varying amounts of CCDs sufficient to cause false-positive results up to 2 kU_A_/L with non-glycosylated recombinant allergens in patients with high levels of anti-CCD IgE antibodies. Clinically irrelevant IgE antibodies against CCDs are found in 20 to 25% of sera of atopic subjects and NRL is not the only “problematic” occupational allergen, also the sIgE measurement of wood allergens [27] is influenced by CCDs. Hemmer and coworkers [26••] recommended in addition to screen for anti-CCD and CCD inhibition easy-to-perform allergen-free dummy CAPs which are useful to identify sera with non-specific background binding. Unfortunately, these allergen-free dummy CAPs are so far not available. A serological work-up for the IgE-mediated latex type-I allergy including at least one CCD screening tool and the recombinant allergens rHev b 1, rHev b 3, rHev b 5, and rHev b 6.01 is highly recommended [17, 18••]. The starting point to evaluate sensitization to NRL is the improved ImmunoCAP test with the Hev b 5-amplified latex extract (k82 “spiked” with rHev b 5) which showed superior sensitivity compared with the results of previously tested negative sera [28, 29]. A recent study of Vandenplas et al. [15] demonstrated that high levels of sIgE to rHev b 5 plus rHev b 6.01 or rHev b 6.02 (≥ 1.46 kU_A_/L; optimal cutoff value based on the highest Youden index) provided the highest positive predictive value (> 90%) for a positive bronchial response to NRL, showing better diagnostic efficiency than the natural rubber latex (k82)-ImmunoCAP. This retrospective study showed that using the sum of sIgE levels to rHev b 5 plus rHev b 6.01 or rHev b 6.02 would be useful in selecting the patients for whom additional diagnostic procedures, such a specific inhalation challenges, would be required for establishing a diagnosis of NRL-induced OA. Nevertheless, none of the subjects with a positive inhalation challenge with NRL gloves and a negative NRL-sIgE result in this series showed IgE reactivity to any of the tested recombinant NRL allergen components, indicating that the assessment of sIgE against the currently available recombinant NRL allergens fails to improve the sensitivity and the negative predictive value of the NRL-sIgE assay.

**Table 1:** Natural rubber latex allergens

Allergen	*Hevea brasiliensis* protein	Clinical relevance
Hev b 1	Rubber elongation factor	Major allergen in SB
Hev b 2	β-1,3-Glucanase	Uncertain^**†**^
Hev b 3	Small rubber particle proteins	Major allergen in SB
Hev b 4	Lecithinase homolog	Minor allergen^**†**^
Hev b 5	Acidic structural protein	Major allergen in HCW and important in SB
Hev b 6.01/6.02	Prohevein/hevein	Major allergen in HCW
Hev b 7	Patatin-like protein (esterase) from latex-B- and C-serum	Minor allergen
Hev b 8	Profilin (actin-binding protein)	Minor allergen
Hev b 9	Enolase	Minor allergen
Hev b 10	Manganese superoxide dismutase (MnSOD)	Minor allergen
Hev b 11	Class I chitinase	Minor allergen
Hev b 12	Non-specific lipid transfer protein type 1 (nsLTP1)	Minor allergen
Hev b 13	Esterase	Uncertain^**†**^
Hev b 14	Hevamine	Minor allergen^**†**^
Hev b 15	Serine protease inhibitor	Minor allergen

*Hev b Hevea brasiliensis*, *SB* spina bifida patients, *HCW* health care workers

^**†**^Not available in recombinant form

**Fig. 1:**
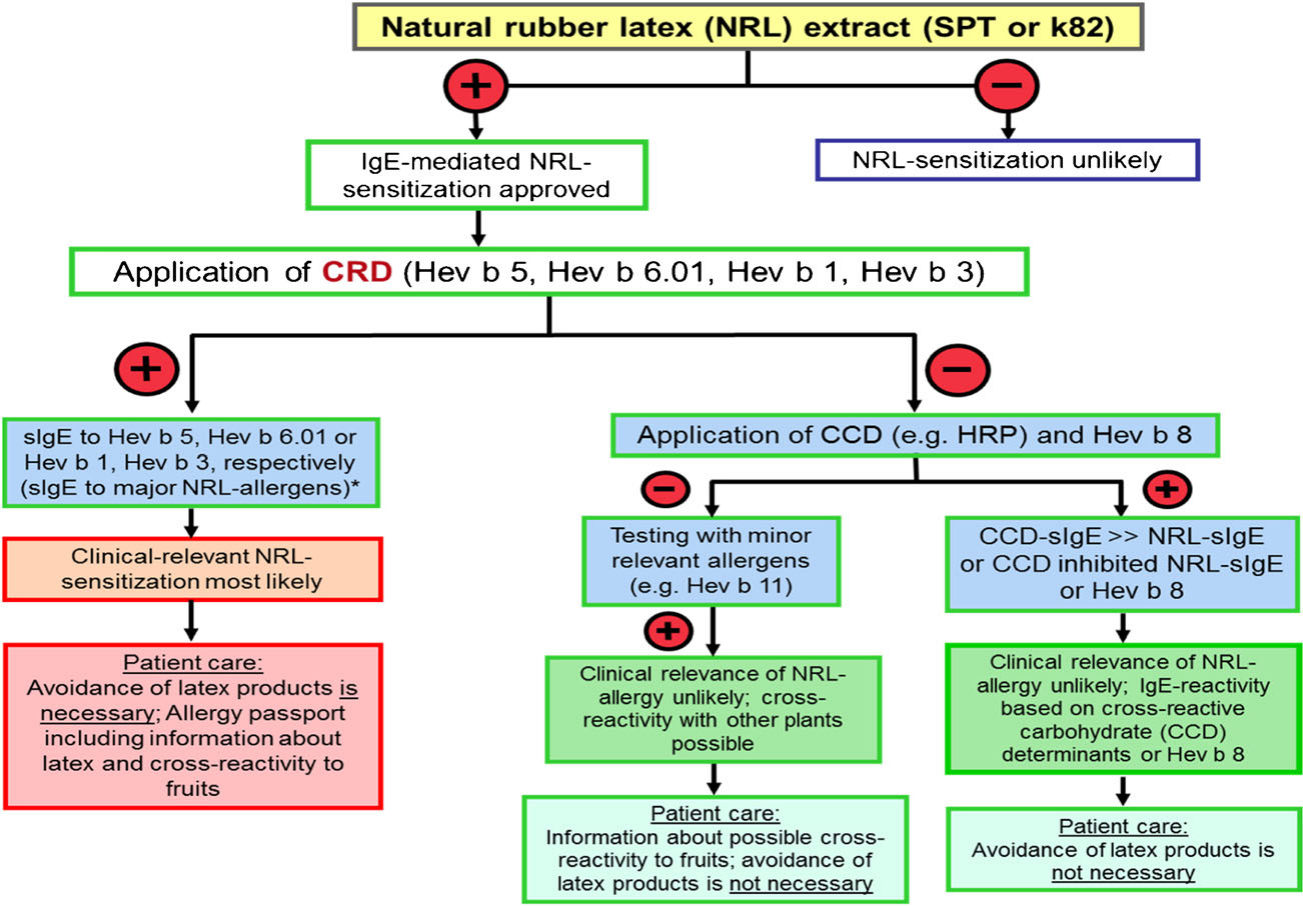
Serological work-up for suspicion of latex type-I allergy or suspicion of polysensitization in patients with specific IgE to latex. This approach is not useful in patients with contact dermatitis and/or protein-contact dermatitis, where additional patch tests including other rubber components (e.g., chemical additives) might be needed (adapted from [18••, 30]). *In the case of positive sIgE to all recombinant Hev b allergens, testing with maltose-binding protein (MBP) ImmunoCAP as a negative control is advisable

Especially in the case of NRL allergy, the in vitro diagnostic tools become more important since in Europe, the “classical” diagnostic tools like NRL extracts for SPT and powdered gloves for workplace-related bronchial challenge tests are no longer readily available; thus, the available recombinant available Hev b allergens combined with CCD tools could be useful in the diagnosis of NRL allergy. The following serological work-up (Fig. 1) might support proper diagnosis in patients with suspected type-I allergy to NRL [18••].

## Cereals

In Western countries, baker’s asthma still remains the most prevalent occupational respiratory disease and most reports indicate that wheat (and also rye) flour proteins are allergens for 60–70% of symptomatic bakers, although other allergens such as enzymes may be involved [31]. SPT with several wheat and rye extracts demonstrated a low sensitivity compared to the specific inhalation challenges [10]. One reason for the insufficient quality of the wheat extracts could be that wheat contains more vegetable proteins than the other two worldwide important cereals, corn and rice [18••], and more than 70 different IgE-binding protein spots have been identified in wheat flour. In contrast to NRL allergy where sera of sensitized health care workers recognized major allergens, several studies have shown that for baker’s asthma, no major allergen could be identified [17, 32, 33]. Twenty-seven wheat (species: *Triticum aestivum*; order: *poales*) allergens are listed so far in the WHO/IUIS Allergen Nomenclature database (www.allergen.org) starting with the wheat profilin (Tri a 12) up to Tri a 45, an elongation factor 1. Tri a 19 (omega-5-gliadin), a 65-kDa seed storage protein, is the best-characterized single wheat component and is involved in wheat-dependent, exercise-induced anaphylaxis (WDEIA) and also for the early childhood type-I wheat allergy [18••, 34]. Tri a 19 is commercially available as a single allergen component but is not relevant for the diagnosis of baker’s asthma [35•]. A number of allergens isolated as native allergen or produced in recombinant form were used in different systems (e.g., singleplex or multiplex, ELISA, immunoblotting) and in various cohorts of bakers with work-related asthma and/or rhinitis symptoms. The results were highly heterogeneous and comparisons between studies are very difficult [32, 36, 37]. In Spanish bakers, wheat lipid transfer protein (LTP) Tri a 14 was described as a major allergen with an overlapping IgE-binding region in peach LTP (Pru p 3) [38, 39]. Armentia et al. [40] performed inhalation challenges with purified Tri a 14 in 27 patients with baker’s asthma from the Spanish region Valladolid and observed in 22 of them a positive bronchial response. A study assessed a panel of 19 recombinant wheat flour allergens and two CCDs with the singleplex technology for sIgE quantification (CAP-FEIA system) in the sera of 101 bakers with occupational allergy from Germany, Spain, and the Netherlands, as well as 29 pollen-sensitized control subjects without occupational exposure but with wheat-sIgE [35•]. The results showed that different α-amylase inhibitors are important allergens for baker’s asthma, but none of the single allergens reached the status of a major allergen. The geographic origin of the subjects was not a significant determinant of the sensitization pattern, and the IgE-binding profile showed a great interindividual variability. Tri a 26 and Tri a 36, relevant wheat allergens in food-allergic patients, were found irrelevant in the diagnosis of baker’s asthma. A combination of sIgE testing to five components (Tri a 27, Tri a 28, tetrameric α-amylase inhibitor CM2 (Tri a 29.02), serine protease inhibitor-like allergen (Tri a 39), and 1-cys-peroxiredoxin (Tri a 32)), produced the highest diagnostic efficiency in receiver operating characteristic analyses, but this was still lower than the determination of sIgE antibodies against the whole wheat or rye flour extracts. In addition, two isoforms of Tri a 14 (Tri a 14.0101, nsLTP 9.1 and Tri a 14.0201, nsLTP 9.7) were tested and both were classified as minor allergens with 11 and 5% positive sIgE-response, respectively in all bakers. Sander et al. [41•] tested Tri a 40.0101 (chloroform/methanol-soluble (CM) 17 protein [alpha-amylase inhibitor], WTAI-CM 17), a further wheat alpha-amylase inhibitor in the same group of bakers and controls and found that this component had only minimal influence on diagnostic sensitivity and failed to improve specificity. Therefore, the first choice for in vitro diagnosis of baker’s asthma is still the determination of allergen sIgE antibodies against the whole wheat flour extracts because of superior diagnostic sensitivity. Bittner et al. [42] reported positive IgE binding to at least 1 of 6 newly identified wheat allergens in 21 (48.8%) of 43 subjects with documented bakers’ asthma who showed negative results with the commercial wheat flour ImmunoCAP. Further studies are necessary in order to determine whether these findings are relevant to the usefulness of these recombinant allergens in diagnosing bakers’ asthma. However, in the future, CRD might help to differentiate between bakers’ asthma, grass pollen allergy, and wheat-induced food allergy, but for this purpose, further single-wheat allergens should be made commercially available.

## Coffee Bean

Dust of green coffee beans is known to be a relevant cause of OR and OA in coffee industry workers [43]. *Coffea arabica* contains arabinogalactan protein complexes which constitute about 15% of the dry weight of the beans. The first coffee bean protein allergen has been isolated, sequenced, and characterized as a class III chitinase with a molecular weight of 32 kDa and listed in the WHO/IUIS database as Cof a 1 [44]. Two cysteine-rich metallothioneins had been identified as novel coffee allergens (Cof a 2 and Cof a 3; 9 and 7 kDa, respectively) [45]. Serum IgE antibodies to at least one of the recombinant allergens Cof a 1, 2, and 3 were detected in 44% of 18 symptomatic coffee workers, while only 11% of them showed sIgE binding to the native green coffee extract. These findings indicate that the only commercially available diagnostic tests based on native extracts of green coffee beans lack sensitivity for establishing an accurate diagnosis in substantial proportion of affected coffee workers, because the natural allergen extracts do not contain sufficient amounts of Cof a 1, 2 and 3. Therefore, the authors recommended the production and application of recombinant coffee allergens for the development of standardized and sensitive diagnostic tools and/or spiking the natural extract with recombinant coffee allergens to improve the diagnostic efficiency of coffee allergy.

## Soybean

Inhalation exposure to soybean (*Glycine max*) dust has been involved in the development of occupational and environmental allergy [46]. The relatively low-molecular-weight proteins concentrated in the soybean hull Gly m 1 (7 kDa, a hydrophobic protein with two isoforms Gly m 1.0101 and Gly m 1.0102) and Gly m 2 (8 kDa, a defensin) were responsible for the asthma attacks during unloading of soybean at the seaports in Spain. In contrast, the allergens involved in OA caused by soybean flour are predominantly high-molecular-weight proteins presented both in soybean hull and flour [47]. Gly m 4, 5, and 6 are currently available as CCD-free recombinant proteins. Further investigation is necessary in order to determine whether these allergens are relevant for the diagnosis of occupational allergy in bakers and other exposed workers.

## Furred Mammals and Derived Products

Mammalian allergens belong to few protein families, including the lipocalins (pheromone-binding proteins), serum albumins, secretoglobins, latherins, cysteine protease inhibitors, and prostatic kallikreins. These allergens are usually present in urine, saliva, and animal dander [48•, 49]. Another large protein family among animal allergens is represented by serum albumins [50], which are present in the blood of animals where they regulate osmotic pressure and transport diverse molecules. Currently, 35 mammalian allergens are listed so far in the WHO/IUIS Allergen Nomenclature database (www.allergen.org) [51•]. Occupational exposure to animal dust is associated with a high risk for the development of respiratory allergy. In Finland, bovine epithelial allergens are responsible for most cases of animal-induced occupational asthma [52]. The predominant allergen in cow dander is Bos d 2, a protein of the lipocalin family [53, 54] (Table 2). This protein family comprises up the majority (> 50%) of the mammalian respiratory allergens [51•]. Bovine serum albumin (BSA; Bos d 6), which is present in bovine plasma, is one of the major allergens affecting patients with food allergies induced by milk and meat. In the context of respiratory allergens, serum albumins are considered as minor allergens without clinical relevance [48•]. However, laboratory workers may be exposed to airborne Bos d 6 as it is widely used in biochemical and immunological assays and two cases of OA have been attributed to inhalation of serum albumin powder (Bos d 6) in laboratory workers [57, 58]. α-Lactalbumin (Bos d 4) and casein (Bos d 8) are typical type I food allergens inducing allergic sensitization via the gastrointestinal tract. A few reports documented occupational airborne exposure to these allergens that induced workplace-related symptoms and occupational allergy [55, 60]. Based on their relevance for cow’s milk-induced allergy, Bos d 4, 6, and 8 are available for sIgE testing (Table 2).

**Table 2:** Mammalian allergens involved in occupational OR/OA

Animal source	Major allergens	Protein family	Main source	Exposed workers (reference)
Cow (*Bos domesticus*)	Bos d 2	Lipocalin	Dander	Dairy farmers [52, 54]
	nBos d 4*	α-Lactalbumin	Milk	Candy and pastry workers [55, 56]
	nBos d 6*	Albumin	Serum	Lab workers [57, 58]
	nBos d 8*	Casein	Milk	Leather tanning [59] Dermatological powder use [55, 60]
Mouse (*Mus musculus*)	Mus m 1**	Lipocalin	Urine	Laboratory animal workers [61]
Rat (*Rattus norvegicus*)	Rat n 1	Lipocalin	Urine	Laboratory animal workers [18••]
Guinea pig (*Cavia porcellus*)	Cav p 1	Lipocalin	Dander, saliva	Laboratory animal workers [18••]
	Cav p 2	Lipocalin	Saliva, dander	

Modified according to [51•]

*Commercially available for component-resolved IgE diagnosis

**Only on immuno-solid-phase allergen chip

Laboratory animal allergy (LAA) is an important occupational disease, and rodents like mice and rats frequently used in animal research are the most common causes of LAA. As common for the most mammalian inhalant allergens, the major allergens in mouse, rat, guinea pig, and rabbit are lipocalins [18••]. Specific IgE determination in the case of LAA based on extracts prepared from epithelia, serum, and/or urine proteins as mixture or alone is useful. Only Mus m 1, the major mouse allergen, is available as a single component on the multiplex test system. Impact of testing individual molecules to determine the severity of symptoms is still unknown.

## Seafood

Occupational respiratory allergy, urticaria and protein-contact dermatitis are serious adverse health outcomes affecting seafood-processing workers. These workers are exposed to airborne seafood particulate matter generated by processing activities that result in the inhalation of airborne allergens [62, 63]. The term seafood includes both fish and shellfish, which are very distinct in evolutionary terms and contain different molecular repertoires of allergens. The shellfish group comprises crustaceans (e.g., crabs, prawns, lobsters) and mollusks (e.g., mussels, squids, scallops) [64•]. The prevalence of OR and OA associated with seafood exposure in epidemiological studies is estimated to be 5–24% [1]. OA is more commonly associated with shellfish (4–36%) than bony fish (2–8%) [65, 66]. Several allergenic proteins have been identified in these different groups: 29 fish allergens and 34 allergens from various crustacean and mollusk species are listed in the WHO/IUIS database so far (www.allergen.org) [64•]. Nineteen fish allergens belong to the parvalbumin family [18••], which is regarded as the fish panallergen and appears to be the major fish allergen [64•]. The first parvalbumin, Gad c 1 from the Baltic cod fish (*Gadus callarias*), was identified in the early seventies, and subsequent cloning and biomolecular studies were performed with the homolog parvalbumin Cad m 1 from *Gadus morhua* (Atlantic cod). Cod parvalbumin is a highly stable protein of the Ca2+-binding EF-hands family with a typical helix-loop-helix structural domain (10–12 kDa) [67] which is mainly found in fish muscle and is relevant both as food and respiratory allergen. Parvalbumin is known to be the cause of important IgE cross-reactivity among fish species, but fish enolase and aldolase have been identified as further important fish allergens [68]. In contrast to ingestion-related food-allergic patients who mainly recognize parvalbumin monomers, sensitized workers with OR and OA appear to recognize higher molecular-weight isoforms [62]. The major shellfish allergens are highly heat-stable proteins, which also cause inhalation exposure and sensitization among workers in the shellfish processing industry leading to asthma and often subsequently food-induced allergy [18••]. Tropomyosin (~ 38 kDa), the major allergen, is the most abundant and heat-stable invertebrate allergen; its amino acid sequence is highly conserved across species and therefore responsible for cross-reactivity between shellfish and other invertebrates. Tropomyosin and also arginine kinase (~ 40 kDa) have been shown to be present as airborne allergen in specific areas during snow crab processing [62]. The availability of individual seafood allergens for sIgE testing is still limited, but two important allergens parvalbumin (rCyp c 1 from *Cyprinus carpio* and rCad c 1 from *Gadus morhua*) and shrimp tropomyosin (rPen a 1 from *Penaeus aztecus* and nPen m 1 from *Penaeus monodon*), as well as prawn arginine kinase (nPen m 2) and sarcoplasmic calcium-binding protein (nPen m 4), are available as singleplex assays and/or on multiplex platforms (microarray techniques). Based on the study of Yang et al. [69] showing that the quantification of tropomyosin-sIgE is superior to SPT with extracts for ingestion-related crustacean allergy, it could be assumed that recombinant allergens may also be relevant to the diagnosis of respiratory allergies in the occupational setting where sensitization results from inhalation exposure.

## Biological Enzymes

Enzymes are proteins that are used as biocatalysts, and they are able to participate in multiple, repeated processes. Therefore, they are efficient ingredients in a variety of industries, including cleaning, food processing, animal feed, textile, paper, and pharmaceuticals (Table 3) [82]. The first cases of respiratory symptoms in detergent workers after inhalation exposure to *Bacillus subtilis*-derived powdered enzymes, alcalase, and maxatase were reported by Flindt and Pepys et al. in 1969 [70, 71]. Out of 25 workers manifesting respiratory symptoms, 20 had a positive SPT induced by enzymatic material prepared from *Bacillus subtilis* and from its spores. These index cases were strong indicators that enzymes were highly allergenic materials and that susceptible workers exposed to these agents were at increased risk of becoming sensitized and developing OA. The majority of microbe-derived enzymes are commonly produced in bacterial microorganisms belonging to *Bacillus* sp. and *Pseudomonas* sp. and fungal organisms such as *Aspergillus* sp., *Streptomyces* sp., and *Trichoderma* sp. Proteases derived from Bacillus, like alcalase and maxatase and savinase, are available as diagnostic tools for sIgE testing. In addition to α-amylase derived from *Aspergillus oryzae*, isolated and denominated as important allergen in baking additives (formerly Asp o II now Asp o 21) [72], β-xylosidase (Asp n 14) [73] and glucoamylase [74] both from *Aspergillus niger* were characterized as relevant allergens in the baking industry. Alpha-amylase, glucoamylase, and also cellulase (derived from *Aspergillus niger*) are available for sIgE testing and used for testing in a study including 433 bakers affected either by OR and/or OA; 299 of them were examined between 1999 and 2001 (group 2000) and 134 between 2009 and 2011 (group 2010) [83]. In total, 30% of affected bakers were sensitized to at least one of the baking enzymes investigated. Serological investigations revealed a significant decline in the rate of sensitization to α-amylase from 26% (in 2000) to 13% (in 2010). Sensitization to glucoamylase and cellulase were determined only in the group 2010 with rates of sensitization 28 and 16%, respectively. Since the early 1990s, phytase derived from *Aspergillus niger* has been increasingly used as an animal feed additive, because this phosphatase enhanced phosphate bioavailability in the gut and reduced the need for phosphate supplements in the feed of monogastric animals. IgE-mediated sensitization to phytase has been reported among workers with work-related respiratory symptoms in a so-called premix factory producing animal feed additives [75]. So far, sIgE testing for phytase is not available. In addition to microbial-derived enzymes, several enzymes used in industry and research are derived from plants. The thiol protease papain produced from the latex of papaya fruit (*Carica papaya*) has a wide variety of applications in the pharmaceutical, cosmetic, and immunochemical industry and as a reagent in biochemical laboratories. The most common sensitization route is inhalation, with high prevalence rates. Several cases of specific airway sensitization caused by papain are verified by a number of case reports and cross-sectional studies [76]. Occupational allergy to papain in exposed workers is associated with OA and/or OR, and sIgE were found in most reported cases. Serological testing is possible with the commercially available nCar p 1. An index case of pepsin-induced OA in a pharmaceutical worker was reported by Cartier et al., and the pepsin sensitization was confirmed by SPT and by sIgE testing. The diagnosis of pepsin-induced OA was confirmed by a positive specific inhalation challenge [77]. For sIgE testing, pepsin is purified from a swine extract. Bromelain, a purified protease of pineapple (*Ananas comosus*), is used, e.g., in pharmaceutical industry and reported as IgE-mediated sensitizer [79]. Bromelain (nAna c 2) is available for sIgE testing but serological tests can yield to false-positive test results due to CCDs [84]. Therefore, the MUXF3 component of bromelain is used as an additional CCD tool and results must be taken with great care when investigating possible allergy to bromelain.

**Table 3:** Biological enzymes involved in occupational OR/OA

Name	Source/production organism	Biological function	Industrial application	Exposed workers	References
Subtilisin (e.g., Alcalase®, Maxatase®, Savinase®)	*Bacillus subtilis* or *Bacillus licheniformis*	Serine protease	Detergent production, food processing	Workers in enzyme production and detergent-manufacturing	[70, 71]
α-Amylase (Asp o 21)	*Aspergillus oryzae*	Catalyzes the hydrolysis of starch into sugars	Food production, baking additive	Baker (mainly)	[72]
β-Xylosidase (Asp n 14)	*Aspergillus niger*	Hydrolysis of (1- > 4)-beta-d-xylans, to remove successive d-xylose residues from the non-reducing termini	Food production, baking additive	Baker (mainly)	[73]
Glucoamylase or amyloglucosidase	*Aspergillus niger*	Starch-breaking enzyme; hydrolyzes terminal 1,4-linked alpha-d-glucose residues successively from non-reducing ends of amylose chains to release free glucose	Food production, baking additive	Baker (mainly)	[74]
Phytase*	*Aspergillus niger*	Phosphatases, breaking down the non-digestible phytic acid	Animal feed supplement	Workers in phytase production	[75]
Papain (n Car p 1)	Papaya fruit (*Carica papaya*)	Thiol protease	Pharmaceutical, cosmetic, and immunochemical industry	Workers in production and application of papain	[76]
Pepsin	Swine extract (Sus)**	Acidic gastric protease; main digestive enzymes	Pepsin powder commonly used in the preparation of F(ab′)2 fragments from antibodies	Laboratory worker, cheese worker	[77, 78]
Bromelain (nAna c 2)	Pineapple (*Ananas comosus*)	Cysteine-endoprotease	Food processing, detergent, and pharmaceutical industry	Workers in food processing industry, laboratory worker	[79, 80]

*Phytase produced in *Trichoderma reesei* has been reported as cause of hypersensitivity pneumonitis in a patient working in a factory producing cattle feed [81]; phytase is so far not commercially available for IgE antibody testing

**Material is purified from a swine extract for IgE antibody testing

## Molds

Exposure to molds is common in various work environments that involve the handling of organic material and exposure to bioaerosols which is the case in agriculture, waste sorting plants, handling with garbage [85], sewage treatment plants, veterinary facilities, and many other workplaces. Exposure to molds can cause allergic rhinitis and asthma, allergic bronchopulmonary mycoses, and hypersensitivity pneumonitis [86–88]. Diagnosis of mold allergy is complicated because of the heterogeneity of the test materials and the decrease in the number of commercial mold extracts for SPT that are currently available [13•, 89]. Therefore, CRD seems to be a suitable alternative [90]. In the WHO/IUIS allergen database, 111 fungal allergens originating from 29 fungal species are listed (www.allergen.org). The most prominent fungal allergen families are proteases, ribosomal proteins, enolases, and dehydrogenases. More than 50% of the WHO/IUIS-classified allergen proteases can be found in molds. Although numerous fungal allergens have been identified, there are currently only eight single mold allergens from three mold genera available for molecular diagnosis. These eight allergens are derived from the species *Alternaria alternata* (rAlt a 1, rAlt a 6), *Aspergillus fumigatus* (rAsp f 1, 2, 3, 4, 6), and *Cladosporium herbarum* (rCla h 8). Available recombinant *Aspergillus fumigatus* (rAsp f) allergens are important because they are valuable tools in the documentation of allergic bronchopulmonary aspergillosis (ABPA) [91]. ABPA is a hypersensitivity lung disease resulting from exposure to *Aspergillus fumigatus* and described, e.g., in garbage workers [92]. Patients with ABPA show elevated specific *Aspergillus fumigatus* serum IgE and frequently elevated IgE to rAsp f 2, rAsp f 4, and rAsp f 6.

## Conclusions

Based on the current data, we would conclude the following:

- Assessment of sIgE reactivity to allergen components has been poorly investigated in the specific field of occupational allergies, with the notable exception of NRL and cereal flour.
- Spiking allergen extracts with recombinant allergens may increase the sensitivity of sIgE assessment against natural extracts (e.g., Hev b 5-amplified latex extract (k82)).
- Assessment of sIgE against occupational allergen components may help to discriminate between different routes of exposure (inhalation vs mucosal exposure), e.g., spina bifida vs health care workers, bakers’ OA from food-induced and exercise-induce allergy
- The determination of sIgE against currently available occupational allergen components failed to increase the diagnostic sensitivity as compared to sIgE antibodies against natural extracts (e.g., NRL induced OA with negative k82), but further identification of allergens could help to establish IgE-mediated sensitization to some allergens encountered at the workplace.
- The determination of sIgE against currently available occupational allergen components from NRL allows for improving the specificity and the positive predictive value for OA.
- For workplace-related allergens like seafood, coffee, soybean, and molds, some characterized allergens are available, but their relevance for occupational sensitization routes should be verified in the future.

## Abbreviations

CCD: Cross-reactive carbohydrate determinants
CRD: Component-resolved diagnosis
HMW: High-molecular-weight
IUIS: International Union of Immunological Societies
LAA: Laboratory animal allergy
LMW: Low-molecular-weight
LTP: Lipid transfer protein
NRL: Natural rubber latex
OA: Occupational asthma
OR: Occupational rhinitis
sIgE: Specific immunoglobulin E antibody
SPT: Skin prick test
WHO: World Health Organization
